# Diagnosis and Treatment of Patients With Focal Segmental Glomerulosclerosis/Steroid-Resistant Nephrotic Syndrome: A Delphi Survey

**DOI:** 10.1016/j.ekir.2022.06.010

**Published:** 2022-06-23

**Authors:** Jürgen Floege, Keisha L. Gibson, Manuel Praga, Jai Radhakrishnan, Heather N. Reich, Michiel F. Schreuder, Jack F. Wetzels, Vladimír Tesař, Marina Vivarelli, Steffen Biechele, Marcello Tonelli

**Affiliations:** 1 Division of Nephrology and Immunology, Rheinisch-Westfälische Technische Hochschule Aachen, Aachen, Germany; 2 Pediatric Nephrology Division, School of Medicine, University of North Carolina, Chapel Hill, North Carolina, USA; 3 Complutense University, Investigation Institute Hospital 12 de Octubre, Madrid, Spain; 4 Division of Nephrology, Columbia University Medical Center, New York, New York, USA; 5 Division of Nephrology, Department of Medicine, University Health Network and University of Toronto, Toronto, Ontario, Canada; 6 Department of Pediatric Nephrology, Amalia Children’s Hospital, Radboud Institute for Molecular Life Sciences, Radboud University Medical Center, Nijmegen, the Netherlands; 7 Department of Nephrology, Radboud University Medical Center, Nijmegen, the Netherlands; 8 Department of Nephrology, Charles University and General University Hospital, Prague, Czech Republic; 9 Division of Nephrology and Dialysis, Department of Pediatric Subspecialties, Bambino Gesù Pediatric Hospital Istituto di Ricerca e Cura a Carattere Scientifico, Rome, Italy; 10 ApotheCom, San Francisco, California, USA; 11 Research Office of the Vice-President, Cumming School of Medicine, University of Calgary, Calgary, Alberta, Canada

**Keywords:** Delphi, focal segmental glomerulosclerosis, nephrotic syndrome, proteinuria, steroid-resistant nephrotic syndrome

## Introduction

Focal segmental glomerulosclerosis (FSGS) is a histopathologic pattern of podocyte injury with several underlying etiologies and is characterized by segmental scarring that involves part of the glomerulus and a subset of glomeruli sampled on biopsy.^1^^,^^2^ In adults, nephrotic syndrome (NS) is often characteristic of primary (or idiopathic) FSGS. In children, steroid-resistantnephrotic syndrome (SRNS) is an indication for kidney biopsy and most commonly associated with FSGS histologically.^3^ Available treatments do not always produce complete remission, and patients who do not achieve remission often progress to chronic kidney failure.^4^, ^5^, ^6^, ^7^

FSGS classification (primary, secondary, genetic, or undetermined cause) and patient-specific factors are used to individualize patient treatment in terms of medications used, dosing, and length of treatment.^1^^,^^8^ International guidelines are available to help nephrologists develop management strategies for patients with FSGS or SRNS,^2^^,^^3^^,^^8^ but the extent to which nephrologists agree with and may apply this guidance is unknown.

The Delphi FSGS and IgA Nephropathy Experts (DEFINE): Physicians study aimed to find consensus on pathophysiology, diagnosis, monitoring, and management of FSGS and IgA nephropathy among nephrologists from Canada, France, Germany, Italy, Spain, the United Kingdom, and the United States. In this 2-round online Delphi survey, agreement with 22 statements about FSGS/SRNS was scored by adult and pediatric nephrologists using a 1 to 9 Likert scale (9 = strongly agree). Moderate versus high consensus were defined as 75% to 89% versus ≥90% of participants scoring 7 to 9 on the Likert scale, respectively. Between November 2020 and April 2021, 207 nephrologists completed round 1, and 158 (76%) nephrologists completed round 2 (Figure S1, Tables S1-S4). Methods and participant characteristics are detailed in the Supplementary Materials.

## Results

In round 1, criteria for high consensus were met for 15 of 22 statements (68%, Table S5), including all 4 pathophysiology statements (statements #1–4) and 11 of 18 diagnosis-focused and treatment-focused statements. Moderate consensus was reached for 5 (23%) statements concerning the treatment of primary or genetic forms of FSGS (statements #10, #13, and #14; Table 1) and the monitoring of patients during initial treatment (statements #20 and #22; Table 1).

**Table 1:** Statements with moderate consensus in round 1 and retested in round 2

Statement No		Round 1 Results				Round 2 Results			
Statements rated by adult nephrologists only		*n*	% Agree	Median	Mean (SD)	*n*	% Agree	Median	Mean (SD)
10	In primary FSGS, immunosuppression is used as initial therapy.	157	82	8	7.5 (1.52)	125^a^	86	8	7.6 (1.23)
10A	In patients with primary FSGS and well-controlled blood pressure, corticosteroids are used as first-line therapy to induce remission.	Revised statement, not tested in round 1				126	88	8	7.8 (1.22)
13	In cases of relapse for steroid-sensitive FSGS (proteinuria >3.5 g/d and serum albumin <30 g/l), a repeat course of corticosteroids is used.	157	89	8	7.6 (1.29)	126	89	8	7.7 (1.17)
13A	In steroid-sensitive FSGS (proteinuria >3.5 g/d and serum albumin <30 g/l), infrequent relapse is treated with a repeat course of corticosteroids.	Revised statement, not tested in round 1				126	87	8	7.9 (1.22)
14	Use of corticosteroids in patients with genetic forms of FSGS is largely ineffective and should be avoided.	157	82	8	7.4 (1.42)	126	81	8	7.4 (1.50)
14A	In adult patients with a documented genetic cause of FSGS, corticosteroids are ineffective.	Revised statement, not tested in round 1				126	86	8	7.7 (1.56)
20	During the initial phase of treatment, monitor the patient every 1–3 mos. If the patient has persistent proteinuria, monitor every 4–6 mos. If the patient becomes nephrotic again, monitor more frequently.	157	88	8	7.7 (1.46)	126	89	8	7.8 (1.14)
20A	In the initial phase of treatment, monitor the patient at least monthly.	Revised statement, not tested in round 1				126	90	8	8.0 (1.12)
20B	For patients in remission, monitor every 3–6 mos thereafter.	Revised statement, not tested in round 1				126	90	8	8.0 (1.06)
Statements rated by pediatric nephrologists only									
22	In children with NS, monitor proteinuria every few days using a dipstick at home. Once in complete remission, monitor proteinuria every 1–4 wks using a dipstick at home (for up to 2 yrs).	50	84	8	7.4 (1.67)	32	81	8	7.6 (1.34)
22A	In children with FSGS/steroid-resistant NS, monitor proteinuria at diagnosis and at least every 3 mos using laboratory testing.	Revised statement, not tested in round 1				32	66	7	6.9 (1.43)
22B	In children with NS, monitor proteinuria daily during induction therapy using a dipstick at home.	Revised statement, not tested in round 1				32	88	8	7.8 (1.48)
22C	In children with NS in complete remission, monitor proteinuria every 1–4 wks, or daily if a respiratory infection occurs, using a dipstick at home for up to 2 yrs.	Revised statement, not tested in round 1				32	91	8	7.9 (1.39)

FSGS, focal segmental glomerulosclerosis; NS, nephrotic syndrome.

Table displays number of respondents, percentage agreement, median and mean (SD) agreement scores for statements that had moderate consensus in round 1 and their updated scores for round 2. Agreement level was scored on a 1–9 Likert scale (1 = strongly disagree, 9 = strongly agree). Consensus was defined as median and mean agreement scores of ≥7 and ≥75% of participants scoring agreement (i.e., 7–9). Statements with 75%–89% agreement were considered to have reached moderate consensus, and statements with ≥90% agreement were considered to have reached high consensus. Based on McNemar’s test, the differences in percentage of agreement between round 1 and round 2 statements were not significant.

a One participant indicated “I do not know” in response to this statement and was excluded from the analysis.

In round 2, revised versions of statements #10, #13, and #14 had very similar agreement levels as their round 1 versions, thereby meeting criteria for moderate consensus (Table 1). Statement #20 on monitoring frequency in adults was revised and divided into 2 statements that met high consensus criteria in round 2, due to small increases in agreement (Table 1). Statement #22 on monitoring frequency in children was revised and divided into 3 statements for round 2 (Table 1). Among the 3 revised statements, only statement #22A did not meet criteria for moderate or high consensus, suggesting that experts may think children’s urine should be monitored by dipstick more frequently.

**Figure 1:**
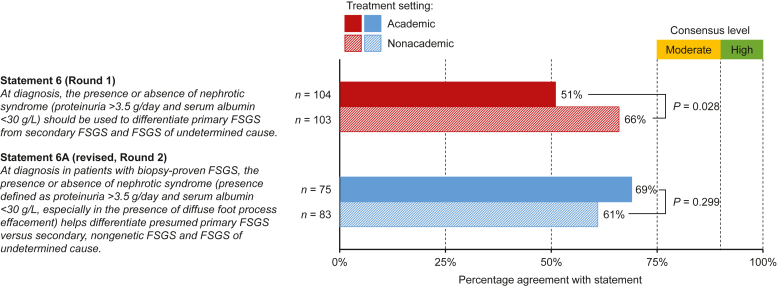
Agreement levels for statement #6 (round 1) and the revised statement #6A (round 2) among participants from academic and nonacademic treatment settings. Statements with 75%–89% agreement were considered to have reached moderate consensus, and statements with ≥90% agreement were considered to have reached high consensus. FSGS, focal segmental glomerulosclerosis.

**Figure 2:**
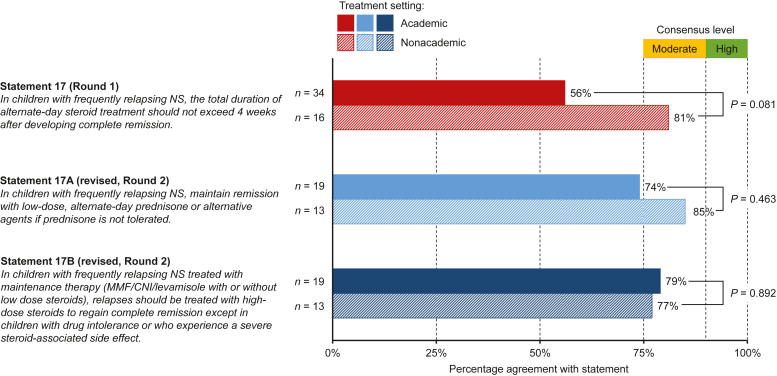
Agreement levels for statement #17 (round 1) and the revised statements #17A and #17B (round 2) among participants from academic and nonacademic treatment settings. Statements with 75%–89% agreement were considered to have reached moderate consensus, and statements with ≥90% agreement were considered to have reached high consensus. CNI, calcineurin inhibitor; MMF, mycophenolate mofetil; NS, nephrotic syndrome.

Of the 22 statements tested in round 1, 2 statements concerning differentiation of primary FSGS from other forms (statement #6) and the optimal duration of steroid treatment in children with frequently relapsing NS (statement #17) did not meet consensus with consensus scores of 58% and 64%, respectively (Figures 1 and 2, Supplementary Table S6). Statement #6 had agreement from 56% of adult nephrologists and 66% of pediatric nephrologists. In round 2, the revised statement, which provided additional details and specificity, did not meet criteria for consensus (65% agreement, Supplementary Figure S2A, Supplementary Table S6). An initial difference in consensus between academic versus nonacademic participants in round 1 (51% vs. 66%, *P* = 0.028; Figure 1) did not persist in round 2 (69% vs. 61%, *P* = 0.299). Statement #17 achieved 64% agreement from pediatric nephrologists in round 1 (Supplementary Figure S2B, Supplementary Table S6). This statement was modified and divided into 2 statements to separate maintenance of remission (statement #17A) from the treatment of relapses during maintenance (statement #17B; Figure 2, Supplementary Figure S2B, Supplementary Table S6). Both revised statements had mean agreement scores of 7.2 (SD 1.70 and 1.88, respectively) and 78% of participants’ agreement (Supplementary Figure S2B, Supplementary Table S6). No significant differences between academic and nonacademic nephrologists were observed for statement #17 in either round (Figure 2).

## Discussion

Overall, these findings revealed a high level of consensus in this multinational group, with 29 of 33 (initial and modified) statements tested meeting moderate or high consensus criteria. High consensus was observed for 4 pathophysiology statements, including the importance of reducing proteinuria to slow disease progression. Statements on treatment decisions also reached high levels of consensus, which may be due to the use of clinical guidelines.^2^^,^^3^^,^^8^

Only 2 statements (#6 and #17) did not meet criteria for consensus in round 1, but agreement levels were still relatively high (>50%). Statements #6/6A focused on whether NS at diagnosis can be used to differentiate primary FSGS from other forms of FSGS (Figure 1). Disagreement with this statement was especially high among adult nephrologists from academic settings. While definitions of FSGS were recently updated,^8^ the lack of consensus observed in this study suggests that many nephrologists are either unaware of or do not agree with these new definitions. The second statement (#17) that did not meet consensus criteria concerned the treatment of pediatric patients with frequently relapsing NS (Figure 2). The revised statements, which differentiated between the initial treatment of pediatric patients with frequently relapsing NS and the treatment of those on maintenance therapy, both achieved moderate consensus. The comments on the original statement (Supplementary Figure S3B) and the agreement with the revised statements suggest that pediatric nephrologists deliver individualized treatment tailored to the patient’s disease status.

Although most consensus statements identified in this Delphi survey aligned with the recently published 2021 KDIGO guideline,^8^ there were notable exceptions. The perception that it is important to reduce proteinuria in patients with FSGS as much as possible was highlighted in several statements from the Delphi panel (statements #7, #19, and #21). Both the 2012 and 2021 KDIGO guidelines do not explicitly state this treatment goal for patients with FSGS, but the 2021 guidelines note that proteinuria quantification “has disease-specific relevance for prognosis and treatment decision-making.”^2^^,^^8^ Similarly, the International Pediatric Nephrology Association SRNS guidelines do not explicitly state a proteinuria reduction goal beyond complete proteinuria remission.^3^ Further, this survey identified several consensus statements without corresponding KDIGO guidance.^8^ These statements described the importance and frequency of patient monitoring (statements #20 and #22) and how to treat relapse in adults with steroid-sensitive FSGS (statement #13).

This study has several limitations. First, the Delphi process was administered only in English and limited to North America and Europe. In addition, female and pediatric nephrologists were underrepresented due to underrepresentation in the recruitment panels. Further, several statements contained more than 1 variable with which participants could disagree. Last, attrition bias is possible, as participants who did not respond in round 2 may have had different viewpoints than those who responded in round 2 (Tables S3 and S4).

In conclusion, the Delphi FSGS and IgA Nephropathy Experts: Physicians Delphi survey identified an overall high level of consensus regarding FSGS/SRNS among adult and pediatric nephrologists. The high levels of consensus reached for most statements and the relatively close alignment between participants’ opinions and current guidelines suggest that perceptions about pathophysiology, the relevance of proteinuria control, and optimal clinical management of patients with FSGS are relatively homogeneous. Future Delphi or survey studies could be used to validate whether this homogeneity persists when evaluated globally. There was relatively less consensus on how best to differentiate primary FSGS from other forms, as well as on the optimal frequency and method of proteinuria monitoring in children with SRNS/FSGS. Future efforts to develop practice guidelines should include more information on how best to differentiate the causes of FSGS.

## Disclosure

JF is employed by Rheinisch-Westfälische Technische Hochschule University of Aachen; has consultancy agreements with Amgen, Bayer, Calliditas, Novo Nordisk, Omeros, Travere Therapeutics, Inc., Vifor, and Visterra; has received honoraria from Amgen, Astellas, Bayer, Calliditas, Novo Nordisk, Omeros, Travere Therapeutics, Inc., Vifor, and Visterra; is a scientific advisor for Calliditas, Omeros, and Travere Therapeutics, Inc.; and is on the speakers’ bureau for Amgen and Vifor. KLG has consulting/advisory commitments with Travere Therapeutics, Inc., Reata Inc., and Aurinia Inc. MP has advisory/speaker agreements with Travere Therapeutics, Inc., Novartis, Alexion, Silence, Glaxo-Smith Kline, and Vifor. JR has received research grants from Travere Therapeutics, Inc.; is on a steering committee for Travere Therapeutics, Inc.; and has consulting/advisory board roles with Angion Biomedica and Travere Therapeutics, Inc. HNR has received consulting fees from Calliditas, Chinook, Novartis, and Travere Therapeutics, Inc.; has received honoraria from Novartis; is an advisor for Novartis and Travere Therapeutics, Inc.; has served as national coordinating investigator for trials by Calliditas and Chinook; has served as an investigator for GN clinical trials by Alnylam, Calliditas, Chemocentryx, Omeros, and Pfizer; and is director of the Glomerulonephritis Fellowship funded by the Louise Fast Foundation. MFS has no disclosures beyond what is listed in the Acknowledgments section. JFW has received grants from Morphosys, Alexion, and Novartis, and has received honoraria from Morphosys, Novartis, and Travere Therapeutics, Inc. VT has served as principal investigator and steering committee member for clinical studies in FSGS supported by Travere Therapeutics, Inc. and has consultancy agreements with AstraZeneca, Boehringer-Ingelheim, Calliditas, Novartis, Omeros, and Travere Therapeutics, Inc. MV is on advisory boards for Apellis, Novartis, Roche, and Travere Therapeutics, Inc.; receives consulting fees from Alexion; and has participated in studies sponsored by Bayer, Novartis, Chemocentrix, and Chinook. This does not influence the content of the present study. SB is employed by ApotheCom, which received funding support from Travere Therapeutics, Inc. for the Delphi FSGS and IgA Nephropathy Experts: Physicians study. MT reports honoraria from AstraZeneca, not related to the topic of the current paper.

## Supplementary Materials

**Supplementary Materials and Methods.**

**Supplementary References.**

**Survey Participants (Description of Study Population).**

**Figure S1.** Flow chart of participant recruitment and retention.

**Figure S2.** Agreement scores for statements #6 and #17 (without consensus).

**Figure S3.** **Themes identified in comments for statements without consensus.**

**Table S1.** Key characteristics of participants

**Table S2.** Additional participant characteristics

**Table S3.** Participant countries and specialties: round 1 and round 2

**Table S4.** Comparison of characteristics between participants and nonparticipants in round 2

**Table S5.** Statements with high consensus in round 1

**Table S6.** Statements without consensus in round 1 and retested in round 2

**Table S7.** Steering committee and research team membership

## Footnotes

Supplementary File (PDF)

**Supplementary Materials and Methods.**
**Supplementary References**.
**Survey Participants (Description of Study Population).**
**Figure S****1****.** Flow chart of participant recruitment and retention. **Figure S****2****.** Agreement scores for statements #6 and #17 (without consensus). **Figure** **S****3****.** Themes identified in comments for statements without consensus. **Table S****1****.** Key characteristics of participants. **Table S****2****.** Additional participant characteristics. **Table S****3****.** Participant countries and specialties: round 1 and round 2. **Table S****4****.** Comparison of characteristics between participants and nonparticipants in round 2. **Table S****5****.** Statements with high consensus in round 1. **Table S****6****.** Statements without consensus in round 1 and retested in round 2. **Table S****7****.** Steering committee and research team membership.
