# Supplementary material for: Diagnosis and Treatment of Patients With Focal Segmental Glomerulosclerosis/Steroid-Resistant Nephrotic Syndrome: A Delphi Survey
Source: Kidney Int Rep. 2022 Jun 23;7(9):2081–5. doi: 10.1016/j.ekir.2022.06.010 (PMC9458994; doi:10.1016/j.ekir.2022.06.010)
Supplement: Supplementary File (PDF) [file mmc1.pdf]

Supplementary Materials

Diagnosis and Treatment of Patients With Focal Segmental Glomerulosclerosis/Steroid-Resistant Nephrotic Syndrome: A Delphi Survey

Authors: Jürgen Floege, Keisha L. Gibson, Manuel Praga, Jai Radhakrishnan, Heather N. Reich, Michiel F. Schreuder, Jack F. Wetzels, Vladimír Tesař, Marina Vivarelli, Steffen Biechele, Marcello Tonelli

Table of Contents

	Page
Materials and Methods	3
Survey Participants (Description of Study Population)	7
Supplementary References	8
Supplementary Figure S1. Flow chart of participant recruitment and retention.	9
Supplementary Figure S2. Agreement scores for statements #6 and #17 (without consensus).	10
Supplementary Figure S3. Themes identified in comments for statements without consensus.	11
Supplementary Table S1. Key characteristics of participants.	12
Supplementary Table S2. Additional participant characteristics.	14
Supplementary Table S3. Participant countries and specialties: round 1 and round 2.	17
Supplementary Table S4. Comparison of characteristics between participants and nonparticipants in round 2.	18
Supplementary Table S5. Statements with high consensus in round 1.	21

Supplementary Table S6. Statements without consensus in round 1 and retested in round 2.	24
Supplementary Table S7. Steering committee and research team membership.	26

Materials and Methods

Study Design

Consensus among nephrologists was evaluated using the Delphi method, which is commonly applied to explore viewpoints and reach consensus on controversial topics.[S1,S2] Consensus is sought over multiple rounds of a Delphi survey by revising statements based on group feedback, as well as giving participants the opportunity to revise their original responses in light of the group response.[S2] The ***De****lphi* ***F****ocal Segmental Glomerulosclerosis (FSGS) &* ***I****gA* ***N****ephropathy (IgAN)* ***E****xperts: Physicians (DEFINE: Physicians)* study was conducted as an online survey in English to allow for a larger and more diverse group of participants than might be possible in face-to-face meetings, and for a wide range of expertise levels to be included without bias introduced by strongly voiced opinions.

The study steering committee (Supplementary Table S7) defined disease pathophysiology, diagnosis, clinical management, treatment decisions, and monitoring as areas of relevance in a virtual workshop meeting and proposed the expert nephrologists with experience in managing FSGS/SRNS to be invited as research team members. For areas of relevance identified by the steering committee, the research team (Supplementary Table S7) developed the initial statements in virtual workshop meetings based on existing clinical guidelines, literature reviews, and the research team's clinical experience. Initial drafts and revisions after Round 1 were further revised, reviewed, and approved by the members of the steering committee and the research team (Table 1, Supplementary Tables S5 and S6). A common set of statements on pathophysiology and disease classification were scored by all participants. Separate statements on clinical management, treatment decisions, and monitoring were developed for the adult vs. pediatric patient populations and scored only by physicians with FSGS/steroid-resistant nephrotic syndrome (SRNS) treatment experience.

In Round 1 of the survey, participants were asked to rate their agreement with each statement using a 1–9 Likert scale (1 = strongly disagree, 9 = strongly agree), with an additional

option to answer, “I do not know.” Agreement was defined as 7–9, and free-text responses were collected from participants rating their agreement ≤6. Consensus was defined as median and mean agreement scores of ≥7 and ≥75% of participants scoring agreement. Statements with 75–89% agreement were considered to have reached moderate consensus, and statements with ≥90% agreement were considered to have reached high consensus (per steering committee decision).

Statements not meeting criteria for high consensus in Round 1 were revised by the research team based on the free-text responses of the participants and approved by the steering committee for Round 2. In Round 2, participants were asked to score their agreement with the revised statements. Participants were also asked to reconsider their initial rating of the original statement after seeing the rating distribution from Round 1, the median score, the percentage of participants agreeing, and their own previous rating. Free-text responses were not collected in Round 2.

Study Population

Nephrologists were recruited from Canada, France, Germany, Italy, Spain, the United Kingdom (UK), and the United States (US), with target participant numbers proportional to the populations of these countries. All participants were required to be board-certified nephrologists, practice in 1 of the 7 target countries, have practiced nephrology for at least 5 years, and have diagnosed or treated ≥2 patients with FSGS/SRNS. Because *DEFINE: Physicians* included a companion Delphi process related to IgAN (reported separately), participants were also required to have diagnosed or treated ≥2 patients with IgAN. Recruitment aimed to include pediatric nephrologists as 30% of participants. To capture treatment patterns from nonacademic and smaller practice settings (community hospital [nonteaching], private office, other), the proportion of participants from academic centers or hospitals was limited to <40% of adult nephrologists (excluding the UK, where all nephrologists were expected to work in academic settings).

Pediatric nephrologists were recruited from academic, non-academic, and smaller practice settings. Participants were recruited by a market research firm (Psyma International Inc., Berwyn, PA, USA, and affiliated partners) from lists of nephrologists validated and updated through standard sources including the American and European Medical Associations, hospital books/directories, medical directories, and verified healthcare internet sites. Participants were not informed of industry funding for this study to avoid bias that might be associated with this information. Participants were remunerated for survey completion.

Ethics Review

The study protocol underwent ethical review by Biomedical Research Alliance of New York and was given exempt status applicable in the US, as this research only included interactions with survey procedures, and response disclosure would not reasonably place the participants at risk. Statements from institutional review boards (IRBs) or forms from relevant national research organizations confirming that IRB approval was not required for this study were collected from non-US countries where the survey was administered. Informed consent was obtained from each participant before survey completion.

Data Collection and Analysis

Participant demographics and characteristics were collected in Round 1. Descriptive statistics were used for participants' characteristics and group responses to each statement. Statistical descriptors calculated included mean and median agreement score and percentage of agreement (score 7–9). The 3 recorded "I do not know" responses were excluded from analysis (Table 1, Supplementary Table S5). Statistical analyses were conducted using Microsoft Excel and SPSS. Percentages of agreement between Round 1 and Round 2 were compared using McNemar's test based on all available data and with a significance threshold of $P = 0.05$.

Agreement levels between participants from academic and nonacademic settings were compared using Pearson’s chi-squared tests.

Survey Participants

Between November 17, 2020, and January 14, 2021, 207 nephrologists participated in Round 1. Participant characteristics for Round 1 are presented in Supplementary Tables S1 and S2. Based on these criteria, the study population included 157 (76%) adult nephrologists and 50 (24%) pediatric nephrologists with a median of 18 years and 17 years of practice in nephrology, respectively. Additional demographic and nephrology-related data (e.g., sources of nephrology-related information) were collected after completing the rating of statements in Round 1. Most participants (92%) indicated that they referred to at least 1 treatment guideline, most commonly the Kidney Disease: Improving Global Outcomes (KDIGO) guideline (88% of adult nephrologists and 66% of pediatric nephrologists). In Round 2 (administered March 29, 2021, to April 13, 2021), 126 of 157 (80%) adult nephrologists and 32 of 50 (64%) pediatric nephrologists from Round 1 completed the survey (Supplementary Tables S3 and S4, Supplementary Figure S1).

References

S1. Belton I, MacDonald A, Wright G, Hamlin I. Improving the practical application of the Delphi method in group-based judgment: a six-step prescription for a well-founded and defensible process. *Technol Forecast Soc Change.* 2019;147:72-82.

S2. Iqbal S, Pipon-Young L. The Delphi method. *Psychologist.* 2009;22:598-600.

Supplementary Figure S1. Flow chart of participant recruitment and retention.

FSGS, focal segmental glomerulosclerosis; IgAN, IgA nephropathy; SRNS, steroid-resistant nephrotic syndrome.

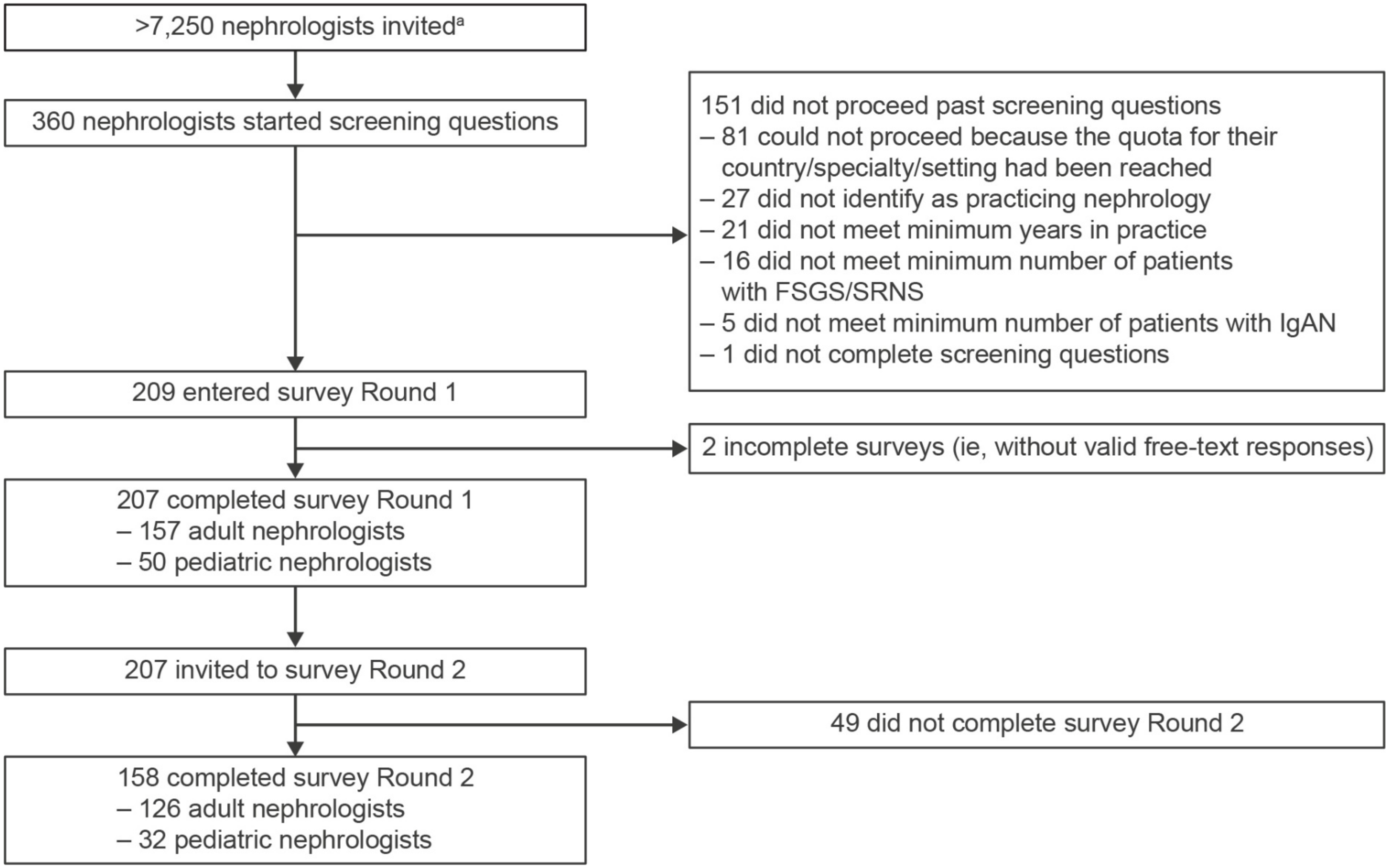

[a] Reasons for not responding to the survey invitation include missing the email invitation, lack of interest in the study topic, unwillingness to participate in an English language survey, and inability to participate at the time (eg, due to time constraints).

Supplementary Figure S2. Agreement scores for statements #6 and #17 (without consensus). (A) Agreement scores and score distribution for statement #6 and revised statement #6A and (B) agreement scores and score distribution for statement #17 and revised statements #17A and #17B. Agreement level was scored on a 1–9 Likert scale (1 = strongly disagree, 9 = strongly agree). Consensus was defined as median and mean agreement scores of ≥7 and ≥75% of participants scoring agreement (i.e., 7–9). Statements with 75–89% agreement were considered to have reached moderate consensus, and statements with ≥90% agreement were considered to have reached high consensus. SD, standard deviation.

A.

	Round 1, *N* = 207	Round 2, *n* = 158
Statement #	6	6A
Agreement (7-9)	58%	65%
Median	7	7
Mean (SD)	6.1 (2.25)	6.8 (1.91)

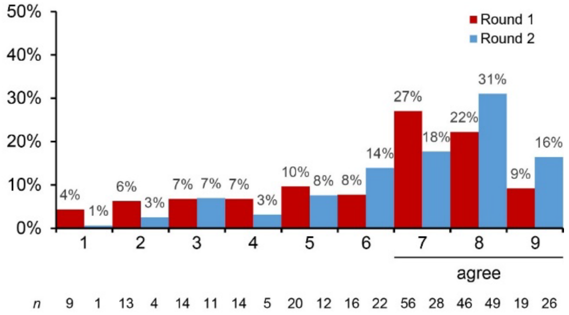

B.

	Round 1, *n* = 50	Round 2, *n* = 32	
Statement #	17	17A	17B
Agreement (7-9)	64%	78%	78%
Median	7	7	8
Mean (SD)	6.3 (2.27)	7.2 (1.70)	7.2 (1.88)

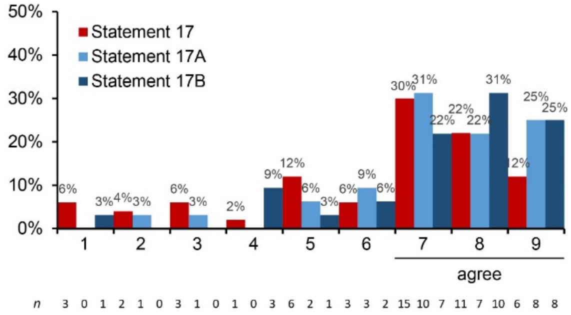

Supplementary Figure S3. Themes identified in comments for statements without consensus. Themes identified in comments by participants with agreement score <7 in round 1 for (A) statement #6 and (B) statement #17. CNI, calcineurin inhibitor; FSGS, focal segmental glomerulosclerosis; MMF, mycophenolate mofetil; NS, nephrotic syndrome; SD, standard deviation.

A.

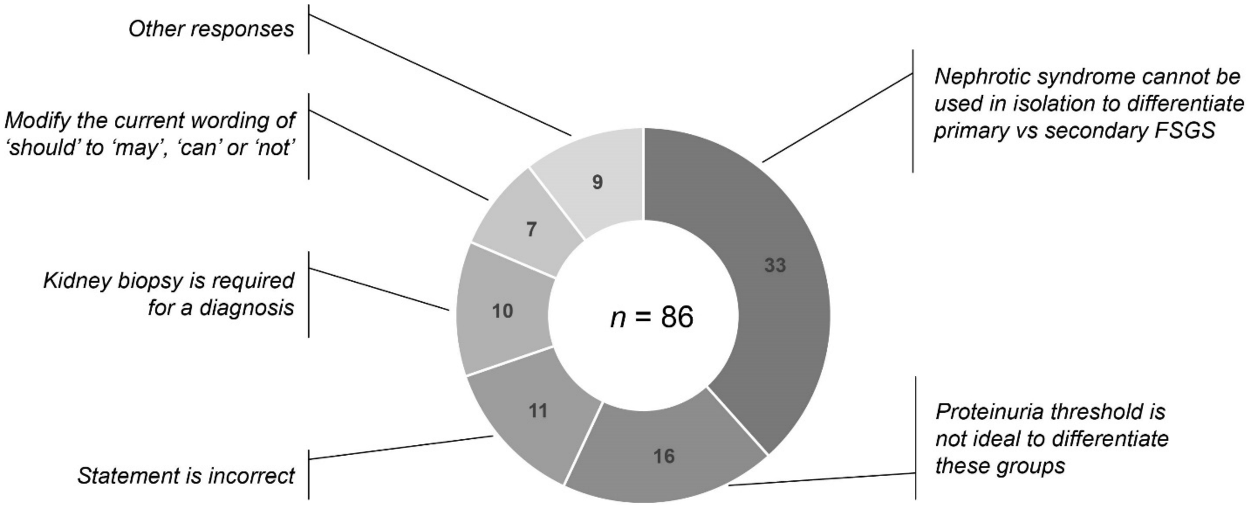

B.

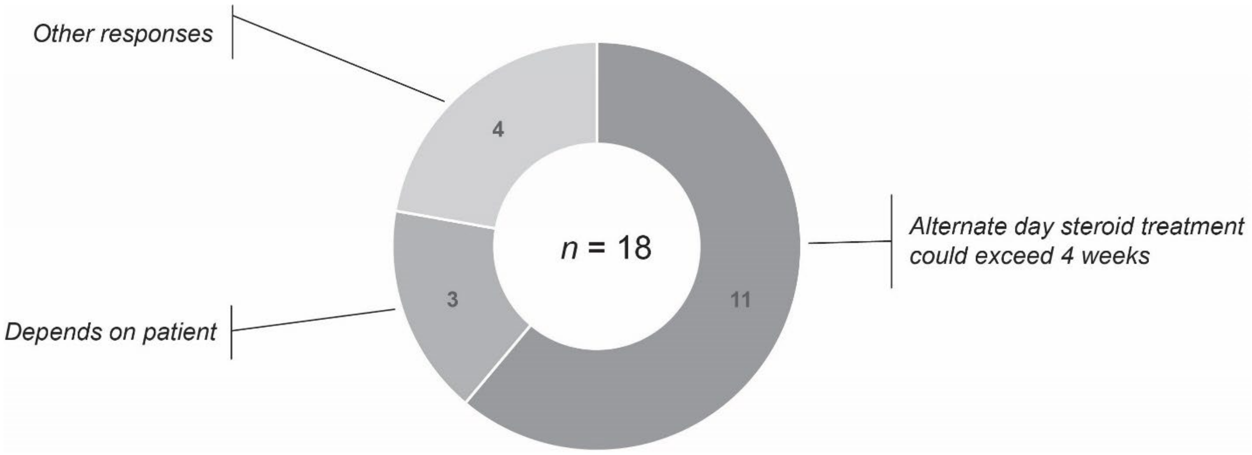

Supplementary Table S1. Key characteristics of participants.

Characteristics	Adult Nephrologists, *n* = 157	Pediatric Nephrologists,[a] *n* = 50	All, *N* = 207
**Characteristics used for participant screening**			
Experience as a practicing nephrologist in years, median (range)	18 (5–49)	17 (5–40)	18 (5–49)
No. of patients with FSGS diagnosed and/or treated in the last 2 years			
Median (IQR)	20 (10–40)	30 (12–60)	20 (10–42)
Mean	31.7	50.6	36.3
Practice setting, *n* (%)			
Academic center or academic hospital	70 (45)	34 (68)	104 (50)
Nonacademic	87 (55)	16 (32)	103 (50)
Community hospital (nonteaching)	37 (24)	7 (14)	44 (21)
Private office	49 (31)	8 (16)	57 (28)
Other	1 (1)	1 (2)	2 (1)
Country of practice, *n* (%)			
United States	69 (44)	26 (52)	95 (46)
Italy	19 (12)	4 (8)	23 (11)
Germany	14 (9)	7 (14)	21 (10)
United Kingdom	15 (10)	5 (10)	20 (10)
France	17 (11)	2 (4)	19 (9)
Spain	13 (8)	5 (10)	18 (9)
Canada	10 (6)	1 (2)	11 (5)

Other characteristics of interest			
Principal investigator in clinical trials,[b] *n* (%)	33 (21)	16 (32)	49 (24)
No. of trials, median (range)	1 (1–5)	2 (1–5)	1 (1–5)
Author/coauthor on nephrology publications in the past 5 years, *n* (%)	90 (57)	36 (72)	126 (61)
No. of publications, median (range)	4.5 (1–165)	4 (1–80)	4 (1–165)
Participants referencing at least 1 clinical guideline when treating patients,[c] *n* (%)	144 (92)	47 (94)	191 (92)
KDIGO	138 (88)	33 (66)	171 (83)
IPNA	7 (4)	27 (54)	34 (16)
ERKNet	17 (11)	13 (26)	30 (14)
AAFP	4 (3)	5 (10)	9 (4)
Other	13 (8)	5 (10)	18 (9)

AAFP, American Academy of Family Physicians; ERKNet, The European Rare Kidney Disease Reference Network; FSGS, focal segmental glomerulosclerosis; IgAN, IgA nephropathy; IPNA, International Pediatric Nephrology Association; IQR, interquartile range; KDIGO, Kidney Disease – Improving Global Outcomes.

[a]Initially, participants were asked to self-identify their specialty (adult nephrology [patients aged ≥18] vs. pediatric nephrology [patients aged 1–18]). To address recruitment challenges, this metric was later changed so that participants with ≥30% pediatric patients were considered qualified to respond to pediatric statements.

[b]Limited to clinical trials enrolling patients with FSGS or IgAN in the last 5 years.

[c]Participants were able to select all that apply.

Supplementary Table S2. Additional participant characteristics.

Characteristic	Adult Nephrologists, *n* = 157	Pediatric Nephrologists *n* = 50	All, *N* = 207
Gender, ***n*** (%)			
Male	130 (83)	40 (80)	170 (82)
Female	25 (16)	8 (16)	33 (16)
Nonbinary	1 (1)	0	1 (<1)
Not disclosed	1 (1)	2 (4)	3 (1)
Ethnic background (US participants only),[a] ***n*** (%)	*n* = 69	*n* = 26	*n* = 95
White or Caucasian	34 (49)	18 (69)	52 (55)
Asian	26 (38)	7 (27)	33 (35)
Multiracial	2 (3)	0	2 (2)
Black or African American	1 (1)	0	1 (1)
Latin-American or Hispanic	0	0	0
Native American or Alaska Native	0	0	0
Native Hawaiian or other Pacific Islander	0	0	0
Other	3 (4)	0	3 (3)
Not disclosed	3 (4)	1 (4)	4 (4)
Presenter at ≥1 congress attended by nephrologists,[a] ***n*** (%)	88 (56)	26 (52)	114 (55)
ASN	43 (27)	14 (28)	57 (28)
ERA-EDTA	36 (23)	11 (22)	47 (23)
NKF	12 (8)	3 (6)	15 (7)

APSN	3 (2)	3 (6)	6 (3)
ANZSN	2 (1)	1 (2)	3 (1)
ISPOR Annual International Meeting	1 (1)	1 (2)	2 (1)
ISPOR Annual European Congress	0	1 (2)	1 (<1)
ISPOR Latin America Conference	0	1 (2)	1 (<1)
AMCP Nexus	0	1 (2)	1 (<1)
Spring Managed Care Forum (NAMCP, AAIHDS, AAMCN)	0	0	0
AMCP Managed Care & Specialty Pharmacy Annual Meeting	0	0	0
Other	31 (20)	9 (18)	40 (19)
Sources of nephrology-related information used,[a] ***n*** (%)			
Articles on UpToDate	122 (78)	31 (62)	153 (74)
Nephrology journals	114 (73)	29 (58)	143 (69)
Nephrology congress attendance	104 (66)	32 (64)	136 (66)
Discussions with other nephrologists	97 (62)	38 (76)	135 (65)
Pharma representatives	44 (28)	13 (26)	57 (28)
Newsletters from nephrology societies	32 (20)	5 (10)	37 (18)
Other	7 (4)	0	7 (3)
Coverage of nephrology-related drug costs covered for most patients, ***n*** (%)			
Public insurance (government insurance or subsidized)	117 (75)	31 (62)	148 (71)
Private insurance (commercial, private, employer, and state health insurance marketplace coverage)	39 (25)	19 (38)	58 (28)

Uninsured or underinsured (and therefore out of pocket)	1 (<1)	0	1 (<1)

AAIHDS, American Association of Integrated Healthcare Delivery Systems; AAMCN, American Association of Managed Care Nurses; AMCP, Academy of Managed Care Pharmacy; ANZSN, Australian and New Zealand Society of Nephrology; APSN, Asian Pacific Society of Nephrology; ASN, American Society of Nephrology; ERA-EDTA, European Renal Association–European Dialysis and Transplant Association; ISPOR, International Society for Pharmacoeconomics and Outcomes Research; NAMCP, National Association of Managed Care Physicians; NKF, National Kidney Foundation; US, United States.

[a]Participants were able to select all that apply.

Supplementary Table S3. Participant countries and specialties: round 1 and round 2.

Country	Specialty	Round 1	Round 2	Percentage of Returning Participants
Canada	Adult nephrology	10	8	80
	Pediatric nephrology	1	1	100
France	Adult nephrology	17	12	71
	Pediatric nephrology	2	2	100
Germany	Adult nephrology	14	10	71
	Pediatric nephrology	7	6	86
Italy	Adult nephrology	19	16	84
	Pediatric nephrology	4	3	75
Spain	Adult nephrology	13	9	69
	Pediatric nephrology	5	2	40
United Kingdom	Adult nephrology	15	10	67
	Pediatric nephrology	5	3	60
United States	Adult nephrology	69	61	88
	Pediatric nephrology	26	15	58
All countries	Adult nephrology	157	126	80
	Pediatric nephrology	50	32	64
Total		207	158	76

Supplementary Table S4. Comparison of characteristics between participants and nonparticipants in round 2.

Characteristics	Adult Nephrologists, *n* = 157			Pediatric Nephrologists,[a] *n* = 50			All, *N* = 207		
	Round 2 Participant *n* = 126	Round 2 Nonparticipant *n* = 31	P value	Round 2 Participant *n* = 32	Round 2 Nonparticipant *n* = 18	P value	Round 2 Participant *n* = 158	Round 2 Nonparticipant *n* = 49	P value
**Characteristics used for participant screening**									
Experience as a practicing nephrologist in years, median (range)	18.5 (5–38)	17 (6–49)	0.533[b]	15 (5–40)	23 (5–38)	0.155[b]	18 (5–40)	18 (5–49)	0.217[b]
No. of patients with FSGS diagnosed and/or treated in the last 2 years									
Median (IQR)	19.5 (10–40)	20 (9–32.5)	0.996[c]	30 (13.75–66.5)	24 (12–54.25)	0.627[c]	20 (10–40)	20 (10–50)	0.847[c]
Mean	31.7	31.8		52.2	47.7		35.8	37.6	
Practice setting, ***n*** (%)									
Academic center or academic hospital	56 (44)	14 (45)	0.943[d]	19 (59)	15 (83)	0.081[d]	75 (47)	29 (59)	0.152[d]

Nonacademic	70 (56)	17 (55)		13 (41)	3 (17)		83 (53)	20 (41)	
Country of practice, ***n*** (%)									
United States	61 (48)	8 (26)	0.276[d]	15 (47)	11 (61)	0.536[d]	76 (48)	19 (39)	0.519[d]
Italy	16 (13)	3 (10)		3 (9)	1 (6)		19 (12)	4 (8)	
Germany	10 (8)	4 (13)		6 (19)	1 (6)		16 (10)	5 (10)	
United Kingdom	10 (8)	5 (16)		3 (9)	2 (11)		13 (8)	7 (14)	
France	12 (10)	5 (16)		2 (6)	0		14 (9)	5 (10)	
Spain	9 (7)	4 (13)		2 (6)	3 (17)		11 (7)	7 (14)	
Canada	8 (6)	2 (6)		1 (3)	0		9 (6)	2 (4)	
**Other characteristics of interest**									
Principal investigator in clinical trials,[e] ***n*** (%)	22 (17)	11 (35)	0.027[d]	11 (34)	5 (28)	0.631[d]	33 (21)	16 (33)	0.090[d]
Author/coauthor on nephrology publications in the past 5 years, ***n*** (%)	66 (52)	24 (77)	0.012[d]	22 (69)	14 (78)	0.495	88 (56)	38 (78)	0.006[d]
Participants referencing at least 1 clinical guideline when treating patients, ***n*** (%)	114 (90)	30 (97)	0.254[d]	29 (91)	18 (100)	0.180[d]	143 (91)	48 (98)	0.088[d]

IQR, interquartile range.

[a]Initially, participants were asked to self-identify their specialty (adult nephrology [patients aged ≥18] vs. pediatric nephrology [patients aged 1–18]). To address recruitment challenges, this metric was later changed so that participants with ≥30% pediatric patients were considered qualified to respond to pediatric statements.

[b]*P* values calculated using Student's t-test.

[c]*P* values calculated using Mann-Whitney U test.

[d]*P* values calculated using Pearson's chi-squared test.

[e]Limited to clinical trials enrolling patients with FSGS or IgAN in the last 5 years.

Supplementary Table S5. Statements with high consensus in round 1.

#	Statement			Round 1 Results	
**Statements rated by all participants**		***n***	**% Agree**	**Median**	**Mean (SD)**
1	Persistently elevated proteinuria is a major adverse prognostic marker in FSGS.	207	97	9	8.4 (0.96)
2	Damage to podocytes and other glomerular cells are amplified by activation of angiotensin and/or endothelin pathways, contributing to high levels of proteinuria and greater risk for progressive kidney injury.	207	92	8	8.0 (1.07)
3	Persistent proteinuria causes tubulointerstitial injury by inducing and amplifying inflammation, fibrosis, and kidney scarring, thereby driving further disease progression.	207	98	8	8.2 (0.82)
4	A close correlation exists between the level of proteinuria and the risk of kidney failure; the higher the proteinuria the higher the risk of kidney failure.	207	96	9	8.2 (1.14)
5	FSGS is a disease characterized by physiological and histological evidence of glomerular injury in a typical pattern of lesions. Due to diverse etiologies and pathogenic mechanisms underlying the FSGS lesion, the disease can be broadly subdivided into four forms: primary FSGS, secondary FSGS, genetic FSGS, and FSGS of undetermined cause.	206[a]	94	8	8.1 (0.97)
**Statements rated by adult nephrologists only**					

7	Patients with non-nephrotic proteinuria and those with nephrotic-range proteinuria but without hypoalbuminemia require optimized supportive therapy with the goal of reducing proteinuria as much as possible.	156[a]	96	8	8.0 (1.13)
8	Patients with nephrotic syndrome (proteinuria >3.5 g/day and serum albumin <30 g/L) exhibit the highest severity of the disease and require a more aggressive therapeutic approach.	157	97	8	8.3 (0.90)
11	For persistent proteinuria, ACE-I/ARBs are used as the basis of optimized supportive maintenance therapy.	157	99	9	8.3 (0.77)
12	In patients with nephrotic syndrome who do not tolerate or are resistant to corticosteroids (proteinuria >3.5 g/day with <50% reduction from baseline despite 16 weeks of steroid use), CNIs may be considered.	157	97	8	8.1 (0.92)
19	In FSGS, the goal of therapy is to reduce proteinuria as much as safely possible in order to preserve kidney function as evidenced by stable or improved GFR.	157	98	9	8.3 (0.87)
**Statements rated by pediatric nephrologists only**					
9	In children, the risk for kidney failure is highly dependent on the response to therapy; patients responsive to steroids are at low risk, patients without complete remission with steroids but responsive to CNIs are at intermediate risk, and patients without remission in response to steroids or CNIs are at highest risk.	50	96	8	8.1 (1.01)

15	In children with NS, oral prednisolone is the standard-of-care initial treatment. Absence of remission after 4 (up to 8) weeks of prednisolone treatment suggests a diagnosis of SRNS.	50	98	8	8.1 (1.06)
16	In children with infrequently relapsing NS, frequently relapsing, and/or steroid-dependent NS, corticosteroids are repeated to treat a relapse.	50	94	8	7.9 (1.20)
18	Treatment options for pediatric patients with steroid-resistant NS are cyclosporine or tacrolimus and RAAS blockade (ACE-I or ARB).	50	96	8	7.9 (1.15)
21	The goal of therapy is to reduce proteinuria as much as safely possible in order to preserve kidney function as evidenced by stable or improved GFR.	50	96	8	8.2 (0.92)

ACE-I, angiotensin-converting enzyme inhibitor; ARB, angiotensin II receptor blocker; CNI, calcineurin inhibitor; GFR, glomerular filtration rate; FSGS, focal segmental glomerulosclerosis; NS, nephrotic syndrome; RAAS, renin-angiotensin-aldosterone system; SD, standard deviation; SRNS, steroid-resistant nephrotic syndrome.

Consensus was defined as median and mean agreement scores of ≥7 and ≥75% of participants scoring agreement (i.e., 7–9).

Statements with ≥90% agreement were considered to have reached high consensus.

[a]One participant indicated “I do not know” in response to this statement and was excluded from the analysis.

Supplementary Table S6. Statements without consensus in round 1 and retested in round 2.

#	Statement	Round 1 Results				Round 2 Results			
**Statements rated by all participants**		***n***	**% Agree**	**Median**	**Mean (SD)**	***n***	**% Agree**	**Median**	**Mean (SD)**
6	At diagnosis, the presence or absence of nephrotic syndrome (proteinuria >3.5 g/day and serum albumin <30 g/L) should be used to differentiate primary FSGS from secondary FSGS and FSGS of undetermined cause.	207	58	7	6.1 (2.25)	158	58	7	6.2 (2.08)
6A	At diagnosis in patients with biopsy-proven FSGS, the presence or absence of nephrotic syndrome (presence defined as proteinuria >3.5 g/day and serum albumin <30 g/L, especially in the presence of diffuse foot process effacement) helps differentiate presumed primary FSGS versus secondary, nongenetic FSGS and FSGS of undetermined cause.	Revised statement, not tested in Round 1				158	65	7	6.8 (1.91)
**Statements rated by pediatric nephrologists only**									
17	In children with frequently relapsing NS, the total duration of alternate-day steroid treatment should not exceed 4 weeks after developing complete remission.	50	64	7	6.3 (2.27)	32	63	7	6.3 (1.96)

17A	In children with frequently relapsing NS, maintain remission with low-dose, alternate-day prednisone or alternative agents if prednisone is not tolerated.	Revised statement, not tested in Round 1	32	78	7	7.2 (1.70)
17B	In children with frequently relapsing NS treated with maintenance therapy (MMF/CNI/levamisole with or without low dose steroids), relapses should be treated with high-dose steroids to regain complete remission except in children with drug intolerance or who experience a severe steroid-associated side effect.	Revised statement, not tested in Round 1	32	78	8	7.2 (1.88)

CNI, calcineurin inhibitor; FSGS, focal segmental glomerulosclerosis; MMF, mycophenolate mofetil; NS, nephrotic syndrome; SD, standard deviation.

Consensus was defined as median and mean agreement scores of ≥7 and ≥75% of participants scoring agreement (i.e., 7–9). Statements with 75–89% agreement were considered to have reached moderate consensus. Based on McNemar's test, the differences in percentage of agreement between Round 1 and Round 2 statements were not significant.

Supplementary Table S7. Steering committee and research team membership.

Steering Committee		
**Name & Affiliation**	**Experience as a practicing nephrologist in years**	**No. of nephrology publications authored/coauthored in the past 5 years**
Jürgen Floege (chair)<br>Division of Nephrology and Immunology, Rheinisch-Westfälische Technische Hochschule Aachen, Aachen, Germany	29	200
Jai Radhakrishnan<br>Division of Nephrology, Columbia University Medical Center, New York, NY, USA	28	51
Heather N. Reich<br>Division of Nephrology, Department of Medicine, University Health Network and University of Toronto, Toronto, ON, Canada	15	50
Vladimír Tesař<br>Department of Nephrology, Charles University and General University Hospital, Prague, Czech Republic	35	125
Marcello Tonelli<br>Research Office of the Vice-President, Cumming School of Medicine, University of Calgary, Calgary, AB, Canada	20	154
Marina Vivarelli<br>Division of Nephrology and Dialysis, Department of Pediatric Subspecialties, Bambino Gesù Pediatric Hospital Istituto di Ricerca e Cura a Carattere Scientifico (IRCCS), Rome, Italy	16	45

Research Team		
**Name & Affiliation**	**Experience as a practicing nephrologist in years**	**No. of nephrology publications authored/coauthored in the past 5 years**
Keisha L. Gibson<br>Pediatric Nephrology Division, School of Medicine, University of North Carolina, Chapel Hill, NC, USA	15	31
Debbie S. Gipson<br>Division of Nephrology, Department of Pediatrics, University of Michigan, Ann Arbor, MI, USA	21	60
Manuel Praga<br>Complutense University, Investigation Institute Hospital 12 de Octubre, Madrid, Spain	39	113
Michiel F. Schreuder<br>Department of Pediatric Nephrology, Amalia Children's Hospital, Radboud Institute for Molecular Life Sciences, Radboud University Medical Center, Nijmegen, The Netherlands	13	52
Jack F. Wetzels<br>Department of Nephrology, Radboud University Medical Center, Nijmegen, The Netherlands	30	92
